# Greater Lumbopelvic Motion Is Associated with Faster Hip Flexion in Soccer Players

**DOI:** 10.3390/sports14020065

**Published:** 2026-02-05

**Authors:** Toshimitsu Ohmine, Akira Iwata, Atsuki Kanayama, Hideyuki Wanaka, Kazuma Senzaki, Mitsuhiro Seo, Keita Sasada, Yoshihiko Kawamoto, Saki Yamamoto, Kenji Doma

**Affiliations:** 1 Graduate School of Comprehensive Rehabilitation, Osaka Prefecture University, Osaka 599-8531, Japan; toshimitsu0605@gmail.com (T.O.); kazuma66gam@gmail.com (K.S.); 2 Department of Rehabilitation Sciences, Division of Physical Therapy, Kansai University of Welfare Sciences, Osaka 582-0026, Japan; 3 Graduate School of Rehabilitation Science, Osaka Metropolitan University, Osaka 599-8531, Japan; kanayama@omu.ac.jp (A.K.); mitsuhiro_s_eo@yahoo.co.jp (M.S.); hchpt_23@yahoo.co.jp (K.S.); yoshihiko19901205@gmail.com (Y.K.); yamamoto-reha@omu.ac.jp (S.Y.); 4 Department of Rehabilitation, Japan Organization of Occupational Health and Safety Kobe Rosai Hospital, Kobe 651-0053, Japan; h.wanaka0531@gmail.com; 5 Sport and Exercise Science, College of Healthcare Sciences, James Cook University, Townsville 4811, Australia; kenji.doma@jcu.edu.au

**Keywords:** lumbopelvic function, soccer players, movement velocity, inertial measurement unit (IMU), hip-flexion task

## Abstract

Faster lower-limb motion is closely related to soccer performance, but the contribution of lumbopelvic motion to achieving it remains unclear. This cross-sectional study aimed to examine whether faster lower-limb motion in soccer players was accompanied by greater lumbopelvic motion. Fifty-one male high school soccer players performed a thigh-raising (hip flexion) task from a standing position at low (approximately 100°/s) and high (approximately 400°/s) speeds. Lumbopelvic motion was measured at the lumbar spinous process (L3). Rotation (LBrot, transverse plane) and flexion–extension (LBf/e, sagittal plane) were derived from the angular velocity. Motions were compared between speeds using the Wilcoxon tests. In the dominant leg, both LBrot (10.2° vs. 8.1°, r = 0.62) and LBf/e (6.4° vs. 5.0°, r = 0.57) were greater at high speed. In the non-dominant leg, both LBrot (11.2° vs. 8.6°, r = 0.49) and LBf/e (6.9° vs. 5.3°, r = 0.62) were also greater at high speed. High-speed trials exhibited consistent movement patterns, whereas low-speed trials did not. These findings suggest that minimizing lumbopelvic motion may not always be optimal for achieving faster lower-limb motion, which may inform coaching and clinical practice regarding the appropriate degree of lumbopelvic motion during lower-limb tasks across movement speeds.

## 1. Introduction

Soccer players require a wide range of abilities, including agility, sprint speed, and kicking speed [1,2]. Sprint speed is affected by the angular velocity of the knee and hip extension on the supporting side and by the angular velocity of the knee and hip flexion on the swinging side [3]. Moreover, ball speed during an instep kick increase with higher angular velocity of knee extension and hip flexion [4]. Consequently, the ability to generate faster lower-limb movements plays a key role in achieving superior soccer performance.

Conceptually, in coaching and sports medicine, it has been hypothesized that faster distal lower limb movements are facilitated when the proximal region (lumbopelvic) remains rigid, with relatively controlled and limited motion [5,6]. This interpretation of “proximal stability” often equates stability with minimal or suppressed motion. Nevertheless, this notion remains largely theoretical, with limited direct experimental validation. In fact, sports performance often requires substantial lumbopelvic motion. For example, Tojima et al. reported greater range of lumbar flexion–extension with increased running speed [7]. In soccer, highly skilled players exhibited a 53% greater rotational range of motion of the trunk than their novice counterparts during an instep kicking manoeuvre, which was moderately correlated with ball speed [8]. Together, these findings suggest that greater, well-coordinated lumbopelvic motion may be associated with superior performance during running and kicking tasks in soccer, thereby challenges the notion that a more rigid proximal region is always required to enhance the movement speed of the distal limbs.

These findings highlight a discrepancy between the theoretical concepts proposed by McGill and Kibler and the empirical data reported by Tojima and Fullenkamp. Despite this apparent conceptual discrepancy, direct experimental clarification remains limited. In recent years, advances in wearable inertial measurement units (IMUs) have enabled the quantification of trunk and pelvic kinematics during lower-limb movements. Several studies have demonstrated the feasibility and validity of using IMUs to assess lumbopelvic motion in both laboratory-based and sport-related tasks [9,10]. In parallel, IMU-based approaches have increasingly been applied to soccer biomechanics to investigate trunk and pelvic kinematics, including during soccer-specific movements such as sprinting, change-of-direction, and kicking actions [11,12,13]. However, these approaches were not specifically designed to determine whether faster lower-limb actions are accompanied by reduced or increased lumbopelvic motion.

This limitation can be attributed to two main reasons. First, in studies such as active straight leg raising [14] and prone hip lifting [15], the change in lumbopelvic position was quantified only between the start and end of the movement, rather than assessing the total magnitude and temporal characteristics of lumbopelvic motion throughout the lower-limb action. As a result, it remains unclear whether the lumbopelvic region was functionally stabilized or dynamically active during the movement. Second, prior investigations examining more dynamic, sport-specific movements, such as sprinting [7] and instep kicking [16], lacked standardization and allowed unrestricted lower-limb motion, further limiting methodological comparability. This lack of standardization complicates the interpretation of whether increased performance is associated with reduced, increased, or optimally coordinated lumbopelvic motion. To address this limitation, we standardized the lower-limb movement range and velocity and quantified the total magnitude of lumbopelvic motion throughout the movement. This approach enables a more direct experimental evaluation of the “proximal stability” versus “dynamic mobility” perspectives under controlled conditions.

In our study, we attempted to address the above-mentioned methodological limitations by measuring and comparing lumbopelvic motion during both slow and fast thigh-raising movements (primarily hip flexion) from a standing position while maintaining the range of motion constant. In so doing, we sought to gain clearer insights into the mechanisms underlying faster lower-limb movements by controlling the movement range and focusing on the total lumbopelvic motion. Although it has been widely suggested that minimizing lumbopelvic motion improves speed, others have challenged this notion by reporting enhanced performance concomitantly with greater lumbopelvic motions. We aimed to support these findings by clarifying whether lumbopelvic motions should increase when a soccer player intends to increase the speed of thigh-raising movements. Thus, we hypothesized that faster lower-limb movements would be accompanied by greater lumbopelvic motion. Specifically, both flexion–extension (sagittal plane) and rotation (transverse plane) of the lumbopelvic motions would be larger under high-speed than under low-speed conditions during the thigh-raising movement examined.

## 2. Materials and Methods

### 2.1. Participants

Seventy-eight male high school soccer players, aged 15–17 years, from three teams, participated in this study. The inclusion criteria for the participants were as follows: (1) hip flexion angles exceeding 100° in both legs, measured with a goniometer in the Thomas test position, defined as the point at which the contralateral hip did not flex; (2) no history of surgery or fractures in the lower limbs or spine; (3) no current pain in the trunk or lower limbs; and (4) no history of low back pain (LBP) within the past three years that suspended competition for more than three days or lasted more than one week. Current LBP was assessed at the time of testing using the Micheli Functional Scale [17] and the Roland–Morris Disability Questionnaire [18]. Participants reporting any degree of LBP, including mild or non-disabling symptoms, were excluded. Information on a history of LBP was obtained using a self-administered questionnaire. Physical maturity (e.g., Tanner stage) was not assessed due to ethical considerations when evaluating pubertal development in minors; however, the sample was relatively homogeneous in age and school grade. This study was conducted in accordance with the ethical principles outlined in the Declaration of Helsinki and was approved by the Human Ethics Committee of Osaka Prefecture University (approval number: 2022-131). Written informed consent for study participation and subsequent publication was obtained from all participants and their parents or legal guardians. Before recruitment, the required sample size for the Wilcoxon signed-rank test was calculated using G*Power 3.1 (Heinrich Heine University, Düsseldorf, Germany) with a statistical power of 0.80, an α = 0.05, and an effect size of 0.50, which is considered a large effect size. This calculation indicated that at least 35 participants were required.

### 2.2. Measures

**Figure 1:**
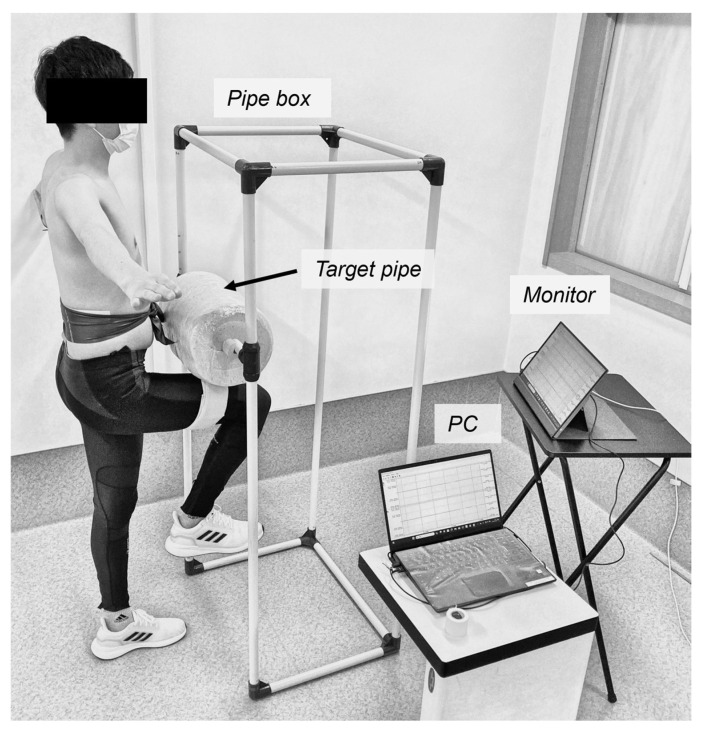
Equipment and measurement environment.

The experimental setup, data acquisition, and data analysis procedures were based on our previous study [19], which quantified lumbopelvic motion during lower-limb movements using inertial measurement units (IMUs). While the experimental setup and speed conditions were identical to those described previously, the present study differed in that comparisons were performed within the same participants between low- and high-speed conditions, rather than between players with and without low back pain. In addition, whereas the previous study analyzed lumbopelvic motion in three directions and a composite measure, the present study focused specifically on the flexion–extension and rotational components of lumbopelvic motion. It should be noted that the axis definition of the thigh-mounted IMU differed from that used in our previous study [19], resulting in an opposite sign of the thigh angular velocity about the *Y*-axis. This difference reflects a difference in the coordinate system definition and was appropriately accounted for during data processing; therefore, it did not affect the interpretation of the results. The experimental setup is illustrated in Figure 1. A pipe box was constructed using plastic pipes to limit and define the range of hip flexion during the thigh-raising task. The target pipe served as a reference point to standardize the participants’ range of thigh motion. The height of the pipe was adjusted so that the participant’s thigh would be parallel to the floor when raised. The monitor provided the participants with real-time feedback on the angular velocity of the thigh movement.

**Figure 2:**
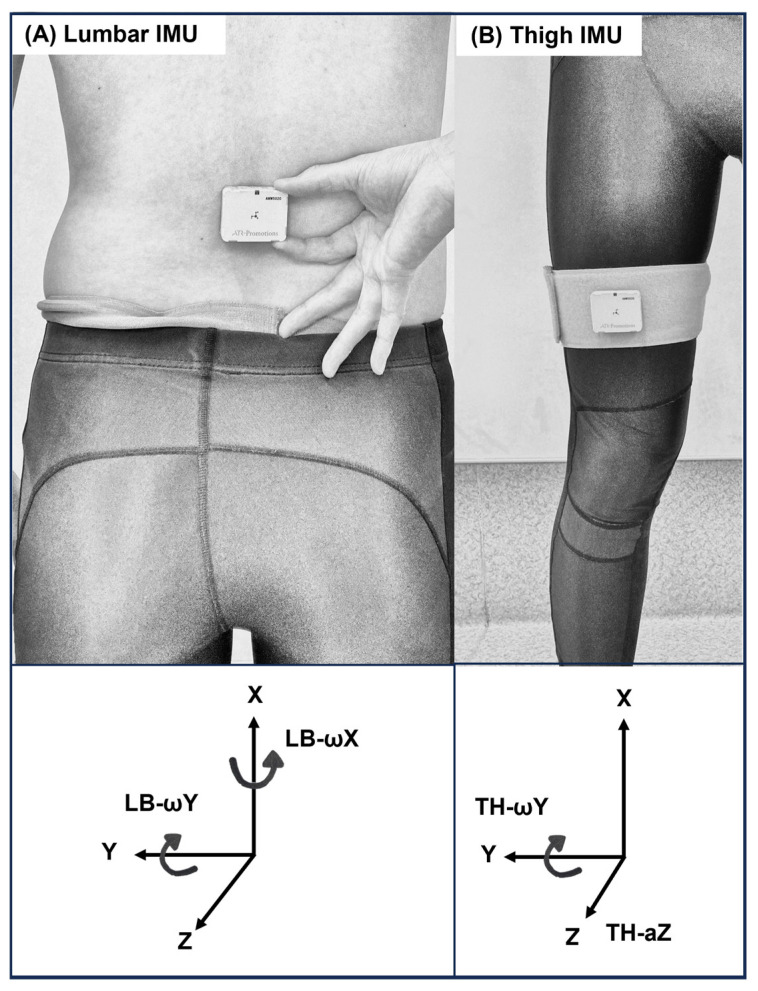
Placement and orientation of the two IMUs. (**A**) Lumbar IMU: Attached above the third lumbar spinous process. The IMU’s axes are oriented with X upward, Y lateral, and Z forward. *LB-ωX* (angular velocity around *X*-axis) indicates lumbar region rotation; *LB-ωY* (angular velocity around *Y*-axis) indicates lumbar region flexion-extension. (**B**) Thigh IMU: Attached 10 cm proximal to the patella. The IMU’s axes are oriented with X upward, Y lateral, and Z forward.TH-ωY (angular velocity around *Y*-axis) indicates thigh-raising velocity; TH-aZ (acceleration along *Z*-axis) detects thigh contact. Arrows indicate the positive directions of the accelerometer (linear axes) and gyroscope (rotational axes). Rotational positive directions follow the right-hand rule. IMU: Inertial measurement unit; ω: angular velocity; LB: lumbar; TH: thigh.

Two IMUs (AMWS020, ATR-Promotions, Kyoto, Japan; ±16 g, ±2000°/s; sampling rate: 500 Hz) were used, each integrating a triaxial accelerometer and a triaxial gyroscope. The sensors were attached to the anterior thigh (10 cm proximal to the patella) and to the spinous process of the third lumbar vertebra; these were defined as the thigh IMU and the lumbar IMU, respectively (Figure 2). The thigh IMU was oriented to measure angular velocity around the *Y*-axis (TH-ωY), representing thigh-raising velocity, and acceleration along the *Z*-axis (TH-aZ), used to detect thigh contact. The lumbar IMU was oriented to measure angular velocity around the *X*-axis (*LB-ωX*), representing lumbar region rotation, and around the *Y*-axis (*LB-ωY*), representing lumbar region flexion–extension (the coordinate axes and positive directions are illustrated in Figure 2). The IMUs data were transmitted wirelessly via Bluetooth to a personal computer (PC; MS-14D2, Micro-Star International, New Taipei City, Taiwan). The *Y*-axis angular velocity waveform from the thigh IMU was displayed simultaneously on the PC and an external monitor, which were connected via a cable. Both IMUs were synchronized prior to measurement.

### 2.3. Design and Procedures

This cross-sectional study compared the amount of lumbopelvic motion during a thigh-raising task performed under low- and high-speed conditions. Each participant performed the task with both legs, and the dominant leg was defined as the preferred kicking leg [20]. The order of the tested leg (dominant vs. non-dominant) and speed condition (low vs. high) was randomized for each participant using simple randomization based on computer-generated random numbers generated in Microsoft Excel. All participants were tested between 09:00 and 12:00 to minimize potential diurnal variation.

Standing in front of the pipe box with both shoulders abducted to 90° to allow visual observation of the lumbopelvic region, participants lifted one thigh to the cushioned target pipe by flexing the hip and knee joints (Figure 1). Participants were instructed to avoid initiating the movement with recoil, maintain the movement speed until contacting the cushion, hold the one-leg standing position for 3 s after contact.

Prior to the measurements, participants completed a standardized warm-up consisting of 5 min of running, followed by familiarization trials. During the familiarization phase, participants practiced the one-leg thigh-raising task using both the dominant and non-dominant legs while monitoring the thigh angular velocity (TH-ωY) displayed on an external monitor as a continuous waveform together with a target angular velocity range indicated by horizontal bars. Visual feedback was provided in real time at a 60 Hz refresh rate, allowing participants to adjust their movements to the target velocities in the low-speed (100°/s) and high-speed (400°/s) conditions. Five practice trials were performed for each condition to ensure adequate familiarization and speed regulation.

After the practice trials, measurements were repeated until three successful attempts were obtained for each condition. No rest interval was provided between repeated trials within the same condition, whereas a 1 min rest period was provided when switching between conditions. A trial was excluded if the movement started with recoil, if the one-leg stance was not maintained for 3 s after contact, or if the peak thigh-raising velocity was outside the target range (50–200°/s for the low-speed condition, 300–500°/s for the high-speed condition).

The high-speed condition (400°/s) was set based on preliminary testing, in which high school soccer players achieved 450–600°/s with maximal effort, to ensure that the target speed was attainable for all participants. This value was also selected to approximate the angular velocity demands of sport-specific movements, as peak hip flexion angular velocities of approximately 375 ± 96°/s have been reported during sprinting [21]. The low-speed condition (100°/s) was set to provide a clearly distinguishable contrast with the high-speed condition, as movements slower than this were observed to be more difficult to control in terms of maintaining a constant angular velocity. For the low-speed condition, a relatively wide tolerance range was applied during data collection because small fluctuations in angular velocity were difficult to visually discriminate in real time using waveform feedback. After data collection, we verified that the low- and high-speed conditions were clearly separated and that the target velocities were successfully achieved using the recorded gyroscope data.

### 2.4. Data Analysis

**Figure 3:**
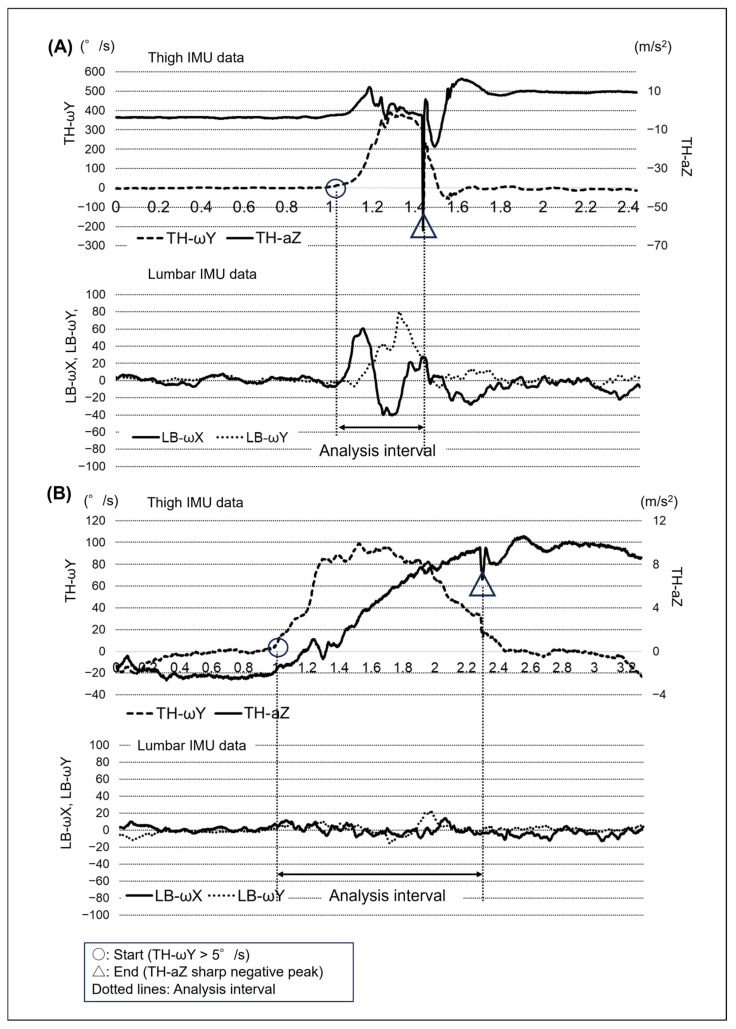
Representative IMU waveforms during High-Speed (**A**) and Low-Speed (**B**) Conditions. Both graphs display thigh IMU data (**upper** panels) and lumbar IMU data (**lower** panels) under high-speed (**A**) and low-speed (**B**) conditions. The start time, indicated by a circle, represents when TH-ωY exceeds 5°/s, marking the initiation of thigh movement. The end time, indicated by a triangle, represents when TH-aZ shows a sharp negative peak, indicating thigh contact and movement termination. The interval between the two dotted lines represents the analysis period. TH, thigh; LB, lumbar; IMU, inertial measurement unit; ω, angular velocity; a, acceleration.

We measured the angular velocity of the lumbar region using a lumbar IMU. As this signal reflects the combined motion of the lumbar spine and pelvis, we interpreted it as a measure of lumbopelvic motion rather than isolated lumbar spine motion. Typical waveforms from the lumbar and thigh IMUs are shown in Figure 3. The analysis interval was defined based on thigh movement data obtained from the thigh IMU. The start time was when the TH-ωY of the thigh IMU exceeded 5°/s (indicating the start of the thigh-raising movement). The end time was when the *Z*-axis acceleration waveform of the thigh IMU (TH-aZ) showed a sharp negative peak (indicating contact of the thigh with the cushion). The subsequent 3 s single-leg stance phase after thigh contact was excluded from the analysis to avoid potential confounding effects of postural stabilization and trunk muscle activation unrelated to the thigh-raising movement. If TH-ωY displayed a value of −5°/s or lower before the initiation of the thigh-raising movement, we judged that the participant had generated a recoil motion involving a small backward displacement of the thigh (i.e., hip extension) and excluded the attempt as a failed trial. The −5°/s threshold for recoil detection was determined based on preliminary testing with synchronized gyroscope signals and high-speed video recordings, which allowed visual confirmation that negative angular velocity ≤ −5°/s corresponded to recoil motion.

The amount of lumbar region motion was calculated using the *X*-axis (*LB-ωX*) and *Y*-axis (*LB-ωY*) angular velocities of the lumbar IMU. Previous studies have demonstrated that *LB-ωX* and *LB-ωY* approximately quantify lumbar region rotational motion and flexion–extension motion, respectively [22]. The IMU signals were processed using the built-in filters specified by the manufacturer. No additional preprocessing (e.g., zero-offset correction, external filtering, or detrending) was applied. Gyroscope drift was not explicitly quantified in the present study. However, we confirmed that the gyroscope output remained close to zero during a brief pre-movement stationary period. In addition, because the integration/analysis window was very short (≤1.3 s), the influence of gyroscope bias accumulation was considered negligible. This assumption is supported by previous studies showing that drift-related errors are limited over short time windows in IMU-based motion analyses [23]. For each attempt, the amount of lumbar region motion was determined by separately integrating the absolute values of *LB-ωX* for rotational motions and *LB-ωY* for flexion–extension motions. The absolute value of angular velocity was integrated to quantify the cumulative magnitude of lumbopelvic motion during the movement. This approach avoids cancellation due to direction reversals, so the resulting values represent total motion magnitude rather than net angular displacement [24]. The time window for integration was defined for each movement event based on sensor signals (Figure 3). The amount of lumbar region transverse plane rotation (LBrot) in one attempt is defined as shown in Equation (1):

(1) $$LBrot=\int_{t1}^{t2}\left|f(LB-\omega X)\right|dt$$

The amount of lumbar region flexion–extension motion (LBf/e) in one attempt is defined as shown in Equation (2):

(2) $$LBf/e=\int_{t1}^{t2}\left|f(LB-\omega Y)\right|dt$$

Data from three successful trials were initially analyzed for all participants, and any failed trial identified after analysis was excluded. All participants successfully completed at least two valid trials for each lower-limb condition (dominant and non-dominant) and both speed conditions (high and low). When all three trials were successful, data from the first and second trials were used to maintain consistency. To confirm the reliability of the two trials per condition, intraclass correlation coefficients (ICC3,2) indicated moderate to good within-subject reliability across speed conditions, movement directions, and legs (ICC3,2 = 0.679–0.836).

### 2.5. Statistical Analysis

We compared the amount of lumbar region motion in the rotational and flexion–extension directions under high-speed and low-speed conditions for both the dominant and non-dominant legs. All variables were confirmed for normality using the Shapiro–Wilk test. The Wilcoxon signed-rank test was used for variables that did not meet normality assumptions. Significance levels were adjusted using the Holm method [25] to control for Type I errors due to multiple comparisons. The significance level was set at *p* < 0.05. Effect size r values were calculated and interpreted according to Cohen’s [26] criteria: 0.10–0.30 was considered small, 0.30–0.50 medium, and ≥0.50 large. All analyses were performed using IBM SPSS Statistics for Windows, version 28.0 (IBM Corp., Armonk, NY, USA).

## 3. Results

The analysis included 51 of 78 soccer players after excluding 15 with current LBP, seven with a history of LBP, two with lower-limb disabilities, and three with incomplete data. Table 1 presents the demographic data of the participants. The achieved thigh-raising angular velocities confirmed clear separation between the speed conditions. Under the high-speed condition, median peak velocities were 395.2°/s [IQR: 380.5–438.3] for the dominant leg and 386.6°/s [373.2–420.0] for the non-dominant leg. Under the low-speed condition, corresponding values were 117.8°/s [97.4–130.8] and 118.0°/s [99.6–134.2], respectively. No overlap in peak angular velocities was observed between the low- and high-speed conditions for either leg.

Figure 3 illustrates the typical waveforms of lumbar region angular velocities in rotation (*LB-ωX*) and flexion–extension (*LB-ωY*) under high-speed (Figure 3A) and low-speed (Figure 3B) conditions. Under high-speed conditions, most participants (46 out of 51; ~90%) rotated their lumbopelvic motion towards the supporting side during the first half of the thigh-raising movement interval and towards the opposite side during the second half. In terms of flexion–extension, most participants (41 out of 51; ~80%) exhibited pronounced lumbopelvic flexion throughout the thigh-raising movement. Conversely, under low-speed conditions, the lumbopelvic motion did not exhibit consistent movement patterns.

**Figure 4:**
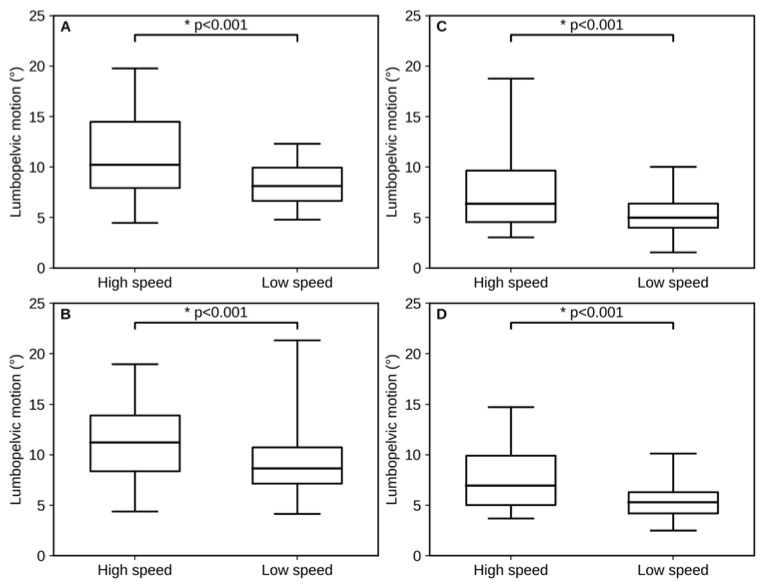
Comparison of lumbopelvic motion during the thigh-raising task under high- and low-speed conditions. (**A**) Lumbopelvic rotation of the dominant leg, (**B**) Lumbopelvic rotation of the non-dominant leg, (**C**) Lumbopelvic flexion–extension of the dominant leg, and (**D**) Lumbopelvic flexion–extension of the non-dominant leg. The box indicates the interquartile range (25th–75th percentiles) and the center line indicates the median. Whiskers indicate the minimum and maximum values.

As shown in Table 2, the amount of lumbar region motion during the thigh-raising movement was significantly greater at high speed than at low speed across all conditions: both rotation and flexion–extension directions for both the dominant and non-dominant legs. All comparisons were statistically significant, and these results remained significant after Holm adjustment for multiple comparisons (all adjusted *p* < 0.05). Figure 4 provides a visual comparison of these results, illustrating the distributions of lumbopelvic rotation and flexion–extension at high and low speeds for both the dominant and non-dominant legs.

## 4. Discussion

Lumbopelvic motion was significantly greater under high-speed than low-speed conditions, both in rotational and flexion–extension, for the dominant and non-dominant legs. Effect sizes ranged from moderate to large (r = 0.49–0.62), indicating that these differences were not only statistically significant but also practically meaningful. Moreover, the movement patterns were consistent in the high-speed condition, whereas no clear pattern was observed in the low-speed condition. These findings support our hypothesis that faster, controlled thigh-raising movement in these male soccer players is accompanied by greater lumbopelvic motion in the transverse and sagittal planes.

From a biomechanical perspective, the finding that lumbopelvic motion increased when the same participants performed faster thigh-raising movements may be interpreted within a proximal-to-distal kinetic-chain framework. In multi-segmental movements, including sports movements, coordinated timing and inter-segmental interactions can facilitate the transfer of both angular momentum [27] and mechanical energy [28] between segments. Accordingly, this within-subject increase in lumbopelvic motion may reflect greater engagement of proximal-to-distal sequencing during rapid thigh motion, potentially involving rapid braking (deceleration) of the proximal segment. However, these mechanistic interpretations (e.g., inter-segmental energy transfer and proximal segment deceleration) were not directly quantified in the present study.

Our findings challenge the clinical hypothesis advocated by Kibler et al. and McGill [5,6], which proposes that efficient distal limb movements occur when the proximal lumbopelvic region exhibits minimal motion. These models, which were developed primarily from clinical and coaching perspectives, emphasize proximal stability as a prerequisite for effective distal mobility. In contrast, the present study showed that greater lumbopelvic motion, particularly flexion–extension and rotation, was associated with faster thigh-raising movements. In addition, future research should confirm these results in other athletes, movements, and perhaps explores potential differences between lumbopelvic motion and the clinical construct of stability. Rather than refuting the stability–mobility framework, our findings suggest that greater lumbopelvic motion appears to be mechanically advantageous for generating distal limb speed in high-speed athletic skills.

We discussed both the rotational and flexion–extension directions of motion to understand why the lumbopelvic motion was greater in the high-speed condition and smaller in the low-speed condition. First, we discuss the rotational direction. Under high-speed conditions, the lumbopelvic region rotates toward the supporting side during the first half of the movement. Previous research has demonstrated that rotational movements of the lumbar during an instep kick transfer energy to the pelvis, which in turn contributes to thigh acceleration through the hip flexion moment [28]. Our findings are consistent with a movement pattern that may reflect a mechanism in which thigh raising is associated with lumbopelvic rotation. In the second half of the movement, the lumbopelvic rotation abruptly decelerated and reversed direction, rotating towards the moving side. A previous study has shown that rapid deceleration of the proximal segment increases the velocity of the distal segment [29,30]. In this context, the proximal segment corresponds to the lumbopelvic region, and the distal segment corresponds to the thigh. This reverse rotation of the lumbopelvic region during the second half of the movement may be observed during faster thigh-raising movements. In contrast, under low-speed conditions, the lumbopelvic region exhibited reduced rotational motion and lacked consistent movement patterns. This may be because, under low-speed conditions, the thigh-raising movement is generated primarily through hip flexion alone, reducing the need for consistent lumbopelvic motion.

Next, we discuss the direction of flexion–extension. Under high-speed thigh-raising movements, the lumbopelvic region exhibited greater flexion than under low-speed conditions. Previous studies have reported that during thigh raising, hip flexion and posterior pelvic tilt occur, with the latter accounting for 13–38% of the total movement [31,32]. Because the IMU attached at L3 captures the combined motion of the lumbar spine and the pelvis, posterior pelvic tilt reduces lumbar lordosis and therefore appears as “lumbopelvic flexion” in the IMU output, rather than representing isolated lumbar spine flexion. Based on this study, the proportions of hip flexion and lumbopelvic flexion during thigh raising are generally considered to remain constant under low-speed thigh-raising movements. However, under high-speed conditions, the contribution of lumbopelvic flexion to thigh raising appeared to increase, thereby enhancing the speed of the thigh-raising movement. This suggests that increased lumbopelvic flexion may substantially contribute to faster thigh-raising movement.

This study has three main limitations. First, the simplified task of lifting the thigh to a parallel position does not fully reflect the complex movements of actual soccer play. This simplification was necessary to standardize the range and speed of the thigh-raising movement; however, the present findings may not be directly generalizable to dynamic sport-specific tasks such as sprinting, cutting, or kicking, which involve more complex whole-body coordination and external constraints. Therefore, future studies should employ experimental tasks that more closely replicate these sport-specific movements. Second, using IMUs instead of a three-dimensional motion analysis system did not provide precise positional information on lumbar and pelvic motions. In addition, measurements obtained from skin-mounted IMUs may be influenced by soft-tissue artefact, sensor alignment error relative to anatomical axes, and potential sensor displacement during movement. Although sensor placement and fixation were carefully standardized, these sources of error cannot be completely eliminated. However, the purpose of this study was not to quantify exact positions but to compare the relative amount of lumbopelvic motion between conditions. In several participants, the IMU-derived angular velocity waveforms showed patterns similar to those obtained from a three-dimensional motion capture system, suggesting that these limitations are unlikely to have substantially affected the interpretation of the relative comparisons. Third, because the participants in this study were male high-school soccer players recruited from only three teams, caution should be exercised when generalizing the findings to other populations, competitive levels, or training environments. In addition, adolescence is a period of ongoing biological maturation and neuromuscular development. Therefore, lumbopelvic control may differ from that of fully mature athletes. Rapid growth during adolescence may influence trunk coordination and movement strategies, which should be considered when interpreting the present findings. Because the present study included only male adolescent players, the findings may not directly generalize to female athletes.

## 5. Conclusions

This study demonstrated that greater lumbopelvic motion was associated with faster thigh raising in male high-school soccer players. Lumbopelvic motion was larger at high speed than at low speed in both rotational and flexion–extension directions, independent of leg dominance. These results indicate that increased lumbopelvic motion was associated with faster thigh-raising movements under controlled task conditions. Despite the limitations of using a simplified movement task and IMUs, these findings highlight the critical role of lumbopelvic motions in achieving rapid lower-limb movements. From a practical perspective, these findings may be applied in training and rehabilitation settings. For example, trunk movement drills and exercises that encourage controlled lumbopelvic rotation or flexion during lower-limb movements may help enhance high-speed movement efficiency. In addition, dynamic pelvic control exercises could be used to improve neuromuscular coordination of the lumbopelvic region. Furthermore, incorporating IMU-based real-time feedback into training may provide athletes with visual information on their lumbopelvic motion, potentially facilitating movement awareness and motor control. This information may also help coaches and trainers provide targeted feedback.

## Figures and Tables

**Table 1:** Demographic data.

Variables	*n* = 51
Age (year) ^a^	16.2 ± 0.6
Years of experience (years) ^a^	7.8 ± 3.4
Position (FW/MF/DF/GK) ^b^	7/19/20/5
Dominant leg (right/left) ^b^	43/8
Height (cm) ^a^	171.1 ± 4.6
Mass (kg) ^a^	60.0 ± 5.7
BMI ^a^	20.5 ± 1.7

BMI: Body mass index, ^a^ Average ± standard deviation, ^b^ Number of data.

**Table 2:** The amount of lumbar region motion during the thigh-raising task.

Variables	High-Speed	Low-Speed	High-Speed	Low-Speed	*p* *	*r* (95%CI)
	Median [IQR]		Mean ± SD			
Dominant leg (°)						
LBrot	10.2 [8.0–14.5]	8.1 [6.7–10.0]	11.0 ± 3.9	8.3 ± 2.0	<0.001	0.62 (0.42–0.76)
LBf/e	6.4 [4.6–9.7]	5.0 [4.0–6.4]	7.3 ± 3.4	5.2 ± 1.9	<0.001	0.57 (0.35–0.73)
Non-dominant leg (°)						
LBrot	11.2 [8.4–13.9]	8.6 [7.2–10.7]	11.4 ± 3.8	9.3 ± 3.5	<0.001	0.49 (0.25–0.67)
LBf/e	6.9 [5.0–10.0]	5.3 [4.2–6.3]	7.5 ± 2.9	5.5 ± 1.8	<0.001	0.62 (0.42–0.76)

IQR: interquartile range, 95%CI: 95% Confidence Interval, SD: standard deviation. LBrot: Amount of lumbar region rotation motion. LBf/e: Amount of lumbar region flexion–extension motion. * *p*-values were adjusted for multiple comparisons using the Holm correction; all adjusted *p* < 0.05.

## Data Availability

The data are not publicly available due to ethical restrictions. The anonymized data are available upon request from the first or corresponding author.

## Abbreviations

The following abbreviations are used in this manuscript:

L3	Third lumbar spinous process
LBrot	Lumbopelvic rotation
LBf/e	Lumbopelvic flexion–extension
LBP	Low back pain
IMU	Inertial measurement unit
TH-ωY	*Y*-axis angular velocity measured by the thigh IMU
TH-aZ	*Z*-axis acceleration measured by the thigh IMU
*LB-ωX*	*X*-axis angular velocity measured by the lumbar IMU
*LB-ωY*	*Y*-axis angular velocity measured by the lumbar IMU
FW	Forward
MF	Midfielder
DF	Defender
GK	Goalkeeper
BMI	Body mass index
